# Vectors of disease at the northern distribution limit of the genus *Dermacentor* in Eurasia: *D. reticulatus* and *D. silvarum*

**DOI:** 10.1007/s10493-020-00533-y

**Published:** 2020-08-19

**Authors:** Franz Rubel, Katharina Brugger, Oxana A. Belova, Ivan S. Kholodilov, Yuliya M. Didyk, Lina Kurzrock, Ana L. García-Pérez, Olaf Kahl

**Affiliations:** 1 Unit for Veterinary Public Health and Epidemiology, University of Veterinary Medicine Vienna, Veterinärplatz 1, 1210 Vienna, Austria; 2 Chumakov Institute of Poliomyelitis and Viral Encephalitides, FSBSI “Chumakov FSC R&D IBP RAS”, Moscow, Russia; 3 Institute of Zoology SAS, Bratislava, Slovakia; 4 Schmalhausen Institute of Zoology NAS of Ukraine, Kiev, Ukraine; 5 IDEXX GmbH, Ludwigsburg, Germany; 6 NEIKER, Department of Animal Health, Vitoria-Gasteiz, Spain; 7 tick-radar GmbH, Berlin, Germany

**Keywords:** Tick-borne diseases, Geographical distribution, Climate adaptation, Köppen–Geiger climate classification

## Abstract

The two ixodid tick species *Dermacentor reticulatus* (Fabricius) and *Dermacentor silvarum* Olenev occur at the northern distribution limit of the genus *Dermacentor* in Eurasia, within the belt of $34{-}60^\circ ~ \hbox {N}$ latitude. Whilst the distribution area of *D. reticulatus* extends from the Atlantic coast of Portugal to Western Siberia, that of *D. silvarum* extends from Western Siberia to the Pacific coast. In Western Siberia, the distribution areas of the two *Dermacentor* species overlap. Although the two tick species are important vectors of disease, detailed information concerning the entire distribution area, climate adaptation, and proven vector competence is still missing. A dataset was compiled, resulting in 2188 georeferenced *D. reticulatus* and 522 *D. silvarum* locations. Up-to-date maps depicting the geographical distribution and climate adaptation of the two *Dermacentor* species are presented. To investigate the climate adaptation of the two tick species, the georeferenced locations were superimposed on a high-resolution map of the Köppen–Geiger climate classification. The frequency distribution of *D. reticulatus* under different climates shows two major peaks related to the following climates: warm temperate with precipitation all year round (57%) and boreal with precipitation all year round (40%). The frequency distribution of *D. silvarum* shows also two major peaks related to boreal climates with precipitation all year round (30%) and boreal winter dry climates (60%). *Dermacentor silvarum* seems to be rather flexible concerning summer temperatures, which can range from cool to hot. In climates with cool summers *D. reticulatus* does not occur, it prefers warm and to a lesser extent hot summers. Lists are given in this paper for cases of proven vector competence for various agents of both *Dermacentor* species. For the first time, the entire distribution areas of *D. reticulatus* and *D. silvarum* were mapped using georeferenced data. Their climate adaptations were quantified by Köppen profiles.

## Introduction

The two ixodid tick species, *Dermacentor reticulatus* (Fabricius) and *Dermacentor silvarum* Olenev are endemic at the northern distribution limit of the genus *Dermacentor* in Eurasia up to approximately $60^\circ ~ \hbox {N}$.

*Dermacentor reticulatus* (in former Russian literature often called *D. pictus*) is the second most often reported tick species after *Ixodes ricinus* in central Europe (Rubel et al. 2014). In sufficiently humid habitats *D. reticulatus* may occur sympatrically with *I. ricinus* or *Ixodes persulcatus* as well as *Haemaphysalis concinna* (Kahl et al. 1992; Hornok and Farkas 2009; Rybár̆ová et al. 2017). The geographical distribution of *D. reticulatus* in Europe was recently mapped by Rubel et al. (2016), whereas a distribution map for the former Soviet Union was published by Kulik and Vinokurova (Kulik and Vinokurova 1983b). The documented distribution of *D. reticulatus* ranges from northern Portugal to Western Siberia. In Europe, distribution is limited to the south by the Mediterranean climatic zone, in which *D. reticulatus* does not occur (exceptions discussed below). In Asia, the cold steppes of Kazakhstan provide a natural barrier for that species. Preferred habitats are alluvial forests where it can survive flooding for certain periods (Nosek 1972). However, it readily colonizes also somewhat drier habitats such as fallow and heathland as well as grassland interspersed with bushes or trees. Remarkable is its occurrence in some urban and suburban areas of big cities such as Berlin (Dautel et al. 2006; Schreiber et al. 2014), Vienna (Leschnik et al. 2012), Budapest (Hornok et al. 2014), Kyiv (Didyk et al. 2017), Moscow (Yankovskaya et al. 2017), and Tomsk (Romanenko et al. 2017). Larvae and nymphs feed on certain rodent species, whereas adults parasitize larger mammals like cattle, deer and dogs but only occasionally bite humans. Whilst only 0.5% of ticks attaching to humans are *D. reticulatus* in Northern Spain (Merino et al. 2005), where *D. reticulatus* is rare, this proportion is above 15% in Western Siberia (Valitskaya et al. 2016). The life cycle lasts 1–2 years or even longer, and the development from egg to unfed adult has to take place in one growing season. Unfed adults are quite long-lived with a life span of up to 3–4 years (Balashov 1972). They are active from late August/September through April/May, interrupted by low temperatures or snow cover in the winter, and enter a behavioural diapause in summer (Belozerov 1982). Oviposition takes place exclusively in spring and the resultant short-lived larvae and nymphs have their main activity periods in July and August, respectively, irrespective of their geographic origin (Kahl and Dautel 2013). Amongst others, *D. reticulatus* is considered the main vector of the Omsk haemorrhagic fever virus in Western Siberia (Růžek et al. 2010) and of *Babesia canis* in Europe (Leschnik et al. 2012; Jongejan et al. 2015). It is also known to be infected with bacteria such as *Rickettsia raoultii* (Barandika et al. 2008).

*Dermacentor silvarum* (formerly sometimes referred to as *D. asiaticus*) is widely distributed in taiga forests in south-eastern Russia (Kulik and Vinokurova 1983a), northern Mongolia (Kiefer et al. 2010), and northeastern and central China (Yu et al. 2011). It can also be found in big cities like the Chinese capital Beijing (Li et al. 2002; Guo et al. 2009). The western distribution limit is in Western Siberia, where *D. silvarum* overlaps with *D. reticulatus*. In the east, *D. silvarum* was documented down to the coast of Sakhalin, the largest island of the Russian Federation in the North Pacific (Kiefer et al. 2010). Its highest abundance has been observed in forest clearings, dry bushland, pastures, and other light-flooded biotopes. In Mongolia, *D. silvarum* has been found in the taiga forests in the northern part of the country and in the forests surrounding the Khentii mountains (C̆erný et al., 2019). Adult females feed mostly on ungulates and wild-boar, but also on hares and hedgehogs. Immatures feed on rodents, hares, and hedgehogs. In Irkutsk, Eastern Siberia, 15% of tick attacks on humans are caused by *D. silvarum* and *D. nuttalli* (Khasnatinov et al. 2016). The life cycle takes approximately one year. The minimum temperatures necessary for development are $8.6\,^\circ \hbox {C}$ for the larvae and $9.7\,^\circ \hbox {C}$ for the nymphs (Beljaeva 1975). Questing adults can be found from late February to early June. They may then enter a behavioural diapause in summer (Belozerov 1982). Adult females engorged after spring enter a reproductive diapause and oviposit in spring of the following year (Balashov 1972). Larvae are active from June to August and nymphs from August to early September (Yu et al. 2010). Amongst others, *D. silvarum* is considered a vector of the tick-borne encephalitis (TBE) virus (Kholodilov et al. 2019) and is also known to be infected with various pathogens.

Maps showing the complete detailed geographical distribution of *D. reticulatus* and *D. silvarum* are missing. In order to compile such maps, a list of georeferenced tick locations is required that was previously collected only for *D. reticulatus* in Europe (Estrada-Peña et al. 2013; Rubel et al. 2016) and *D. silvarum* in China (Zhang et al. 2019). Large areas of eastern Europe and Asia (Ukraine, Belarus, Kazakhstan, Mongolia, and Russia) have not or only sparsely been covered by georeferenced data. Therefore, the current geographical distribution limits of these species are not well documented. The *Dermacentor* maps presented here should help to fill this serious gap. For this purpose not only historical datasets from Ukraine and Russia were digitized, but numerous recently published tick locations from the entire distribution area in Eurasia were compiled. This renders not only distinctly improved *D. reticulatus* and *D. silvarum* maps, but also a database, usable for modelling of tick habitats and the occurrence of tick-borne diseases vectored by these species.

Particular attention was paid to the climate adaptations of the two herein investigated tick species. As recently done for depicting the distribution of the soft ticks *Argas miniatus* and *Argas persicus* (Muñoz-Leal et al. 2018) as well as the Eurasian hard tick *H. concinna* (Rubel et al. 2018), georeferenced tick sampling sites were superimposed on climate maps.

## Materials and methods

Knowledge on the geographical distribution of *D. reticulatus* and *D. silvarum* in Eurasia is based on the existing datasets of Rubel et al. (2016) with 1207 *D. reticulatus* locations, and 181 *D. silvarum* locations compiled by Zhang et al. (2019). Two locations were removed from the dataset published by Rubel et al. (2016). The first was in southern Portugal, where according to Santos-Silva et al. (2011) no *D. reticulatus* occur. The second location contained a wrong coordinate. The original dataset of Zhang et al. (2019) comprises geographical coordinates of 404 (221 without duplicates) *D. silvarum* locations in China collected during the period 1954–2017. These tick locations were classified into four different levels according to their geographic scales and administrative levels (1=provincial, 2=prefectural, 3=county, 4=township or finer). A total of 41 locations was excluded, which reduces the number of *D. silvarum* locations taken from Zhang et al. (2019) down to 180. In order to supplement these already existing two data sets, a comprehensive literature research was carried out. It refers mainly to those studies in which georeferenced findings were documented. Exceptions were made when sufficient information on the locations or printed maps were available as a basis for digitization. According to Table 1 the following numbers of *D. reticulatus* locations were incorporated: 10 in Austria, 2 in Bosnia and Herzegovina, 21 in Croatia, 17 in France, 31 in Germany, 9 in Hungary, 8 in Italy, 45 in Kazakhstan, 10 in Moldova, 3 in the Netherlands, 36 in Poland, 11 in Portugal, 219 in Russia, 2 in Serbia, 413 in the former Soviet Union, 18 in Spain, 1 in Switzerland, and 120 in Ukraine. The digitized *D. silvarum* locations are listed in Table 2 and composed as follows: 214 in China, 10 in Mongolia, 85 in Russia, and 213 in the former Soviet Union.

As depicted in the Tables 1 and 2, the majority of the references considered were published during the period 2010–2019. Contrary to an increasing number of *Dermacentor* studies in countries of the European Union, sparsely populated regions of Eurasia are not sufficiently covered by existing studies. Thus, data or handdrawn maps from older studies are still relevant even if they had to be digitized before they could be added to the new *Dermacentor* maps. These include above all the two maps by Kulik and Vinokurova (1983a, b), without which a good coverage of the countries of the former Soviet Union would not be possible. The same is true for Ukraine, for which Hightower et al. (2014) compiled serveral tick maps in his master thesis. These tick maps are based on data of the period 1940–2008 provided by the CSES (Central Sanitary Epidemiological Station) bacterial archives in Kyiv, Ukraine (Hightower 2012). Some German *Dermacentor* locations mapped by Pluta et al. (2010) are given in detail in the PhD thesis of Pluta (2011). On the other hand, the detailed map of the Russian Tula region (Kozlova et al. 2016) contained too many *D. reticulatus* locations for the maps compiled here, so that only 30 randomly selected locations of the period 2003–2013 were used.

**Table 1:** Number, accuracy (low, medium, high and unspecified), and country of georeferenced *Dermacentor reticulatus* sampling sites compiled in this study

No.	Acc.	Country	References
10	m	Austria	Hodžić et al. (2017b)
1	l	Bosnia	Krčmar et al. (2014)
1	h	Bosnia	Hodžić et al. (2017a)
2	h	Croatia	Radzijevskaja et al. (2015)
19	h	Croatia	Krčmar (2019)
7	m	France	René-Martellet et al. (2015)
2	m	France	Michelet et al. (2016)
8	l	France	LK
14	l	Germany	Pluta et al. (2010)
10	l	Germany	Schreiber et al. (2014)
4	h	Germany	Kohn et al. (2019)
3	h	Germany	OK
9	m	Hungary	Hornok et al. (2014)
1	h	Italy	Genchi et al. (2015)
6	h	Italy	Olivieri et al. (2016)
1	h	Italy	Olivieri et al. (2017)
45	l	Kazakhstan	Amirova et al. (1989)
10	l	Moldova	Movila et al. (2006)
3	h	Netherlands	Hofmeester et al. (2016)
23	l	Poland	Zygner et al. (2009)
13	h	Poland	Kubiak et al. (2018)
11	l	Portugal	Santos-Silva et al. (2011)
1	l	Russia	Ulyanova et al. (1969)
1	l	Russia	Matushchenko et al. (1993)
3	l	Russia	Evstaf’ev (2001)
2	l	Russia	Filippova and Stekolnikov (2007)
2	l	Russia	Shpynov et al. (2008)
4	m	Russia	Gubeidullina et al. (2009)
2	l	Russia	Samoilenko et al. (2011)
26	l	Russia	Tohov et al. (2013)
13	h	Russia	Belova et al. (2014)
1	m	Russia	Dedkov et al. (2014)
8	l	Russia	Kholodilov et al. (2014)
50	l	Russia	Obert et al. (2015)
6	l	Russia	Shchuchinova et al. (2015)
4	l	Russia	Shamsutdinov et al. (2015)
4	l	Russia	Volkov and Bessolytsina (2015)
30	l	Russia	Kozlova et al. (2016)
22	l	Russia	Norkina (2016)
1	l	Russia	Milintsevich et al. (2016)
7	h	Russia	Romanenko et al. (2017)
22	m	Russia	Yankovskaya et al. (2017)
1	l	Russia	Kirillova and Kirillov (2018)
3	l	Russia	Korzikov et al. (2018)
4	m	Russia	Bakhtushkina (2019)
2	h	Russia	Turebekov et al. (2019)
1	l	Serbia	Jurišić et al. (2012)
1	l	Serbia	Pavlović et al. (2016)
5	h	Slovakia	Radzijevskaja et al. (2015)
413	l	Soviet Union	Kulik and Vinokurova (1983b)
12	h	Spain	Barandika et al. (2011)
5	l	Spain	LK
1	h	Spain	Remesar et al. (2019)
1	l	Switzerland	Eichenberger et al. (2015)
120	l	Ukraine	Hightower et al. (2014)
1207	u	European Countr.	Rubel et al. (2016)
2188	–	Total	

Sites not referenced here were provided by the authors OK (3 sites in Germany) and LK (5 sites in Spain, 8 sites in France)

**Table 2:** Number, accuracy (low, medium, high and unspecified), and country of georeferenced *Dermacentor silvarum* sampling sites compiled in this study

No.	Acc.	Country	References
1	h	China	Yu et al. (2010)
13	h	China	Jiang et al. (2011)
3	l	China	Liu et al. (2016)
8	l	China	Sun et al. (2017)
2	m	China	Han et al. (2018)
2	l	China	Jia et al. (2018)
1	l	China	Wang et al. (2018)
2	l	China	Zhang et al. (2018)
1	l	China	Meng et al. (2019)
1	l	China	Zhao et al. (2019)
180	l	China	Zhang et al. (2019)
10	l	Mongolia	Hightower et al. (2012)
6	l	Russia	Kolonin et al. (1984)
2	l	Russia	Filippova and Apanaskevich (2005)
2	l	Russia	Danchinova et al. (2007)
3	l	Russia	Balakhonov et al. (2012)
1	l	Russia	Danchuk et al. (2012)
1	m	Russia	Bolotova et al. (2014)
2	m	Russia	Rar et al. (2014)
1	l	Russia	Gordeiko (2015)
1	l	Russia	Kurganova et al. (2015)
19	l	Russia	Obert et al. (2015)
7	l	Russia	Shchuchinova et al. (2015)
1	l	Russia	Zvereva et al. (2015)
2	m	Russia	Bogdanov et al. (2017)
1	m	Russia	Lubova et al. (2017)
7	l	Russia	Chistyakova et al. (2018)
4	l	Russia	Igolkina et al. (2018)
2	l	Russia	Leonova et al. (2018)
4	m	Russia	Pukhovskaya et al. (2018)
4	h	Russia	Seryodkin et al. (2018)
14	h	Russia	Kholodilov et al. (2019)
1	l	Russia	Doroshchenko et al. (2019)
213	l	Soviet Union	Kulik and Vinokurova (1983a)
522	–	Total	

Digitized locations, of course, are generally of lower accuracy than locations described by geographical coordinates determined by GPS in the field. To provide evidence of this, accuracy measures were given for all data referenced in Tables 1 and 2 in accordance with the scheme applied by Rubel et al. (2014, 2016, 2018). It is distinguished between high (h), medium (m), low (l) and unspecified (u) accuracies. The latter has been applied here only to the transnational record of Rubel et al. (2016) that contains tick locations of all accuracy levels. A high accuracy ($\pm \, 0.1\, \hbox {km}$) was allocated to coordinates given in degrees, minutes and seconds or in decimal degrees with at least 4–5 relevant decimal places. A medium accuracy ($\pm \, 1\, \hbox {km}$) was assumed for coordinates given in degrees and minutes or in decimal degrees with at least 2–3 relevant decimal places. A medium accuracy was also assumed for ticks collected from animals or humans and for coordinates digitized from local maps. Coordinates digitized from regional maps were classified as low-accuracy data ($\pm \, 10\, \hbox {km}$). After data collection, homogenization of the associated geographical coordinates (conversion to decimal degrees with 4 digits), and homogenization of the accuracy measures, the final dataset was compiled by eliminating further duplicate entries.

To visualize the geographical distribution of *D. reticulatus* and *D. silvarum*, the georeferenced locations were plotted on terrain maps (OpenStreetMap contributors 2017). They show the distribution patterns of the two tick species determined by continental-scale mountain ranges like the Himalayas and surrounding steppes and deserts. The latter were also depicted in a second type of maps, where the tick locations were plotted on climate maps. Therefore, new global maps of the Köppen–Geiger climate classification (Rubel and Kottek 2010) were calculated from the latest version of temperature fields provided by the Climatic Research Unit (CRU) of the University of East Anglia and precipitation fields from the Global Precipitation Climatology Centre (GPCC) at the German Weather Service. Compared to Köppen–Geiger maps used in the previous study by Rubel et al. (2018), three times more Chinese precipitation measurements (Andreas Becker (GPCC), personal communication) lead to a significantly improved climate classification of important *D. silvarum* distribution areas. Generally, the Köppen–Geiger climate classification is based on 31 climate classes described by a three-letter code. The first letter distinguishes between different types of vegetation of the equatorial zone (A), the arid zone (B), the warm temperate zone (C), the boreal or snow zone (D), and the polar or ice zone (E). The second letter in the classification considers precipitation (e.g. Cf for warm temperate and precipitation all year round) and the third letter considers air temperature (e.g. Cfb warm temperate, precipitation all year round and warm summer).

The climate map (version December 2018) is provided on http://koeppen-geiger.vu-wien.ac.at/present.htm together with the underlying digital data and an R code (R Development Core Team 2019) for reading and visualization. The gridded climate classification is available with a spatial resolution of 5 arcmin and representative for the 25-year period 1986–2010. It was calculated from downscaled, i.e. disaggregated (Rubel et al. 2017), CRU V4.03 temperature and GPCC V8 precipitation fields as described by Kottek et al. (2006). With this dataset, each tick location can be related to a specific climate class in order to calculate a histogram. Recent application of this so-called Köppen profile were, for example, presented by Grímsson et al. (2018) and Rubel et al. (2018).

## Results and discussion

**Fig. 1:**
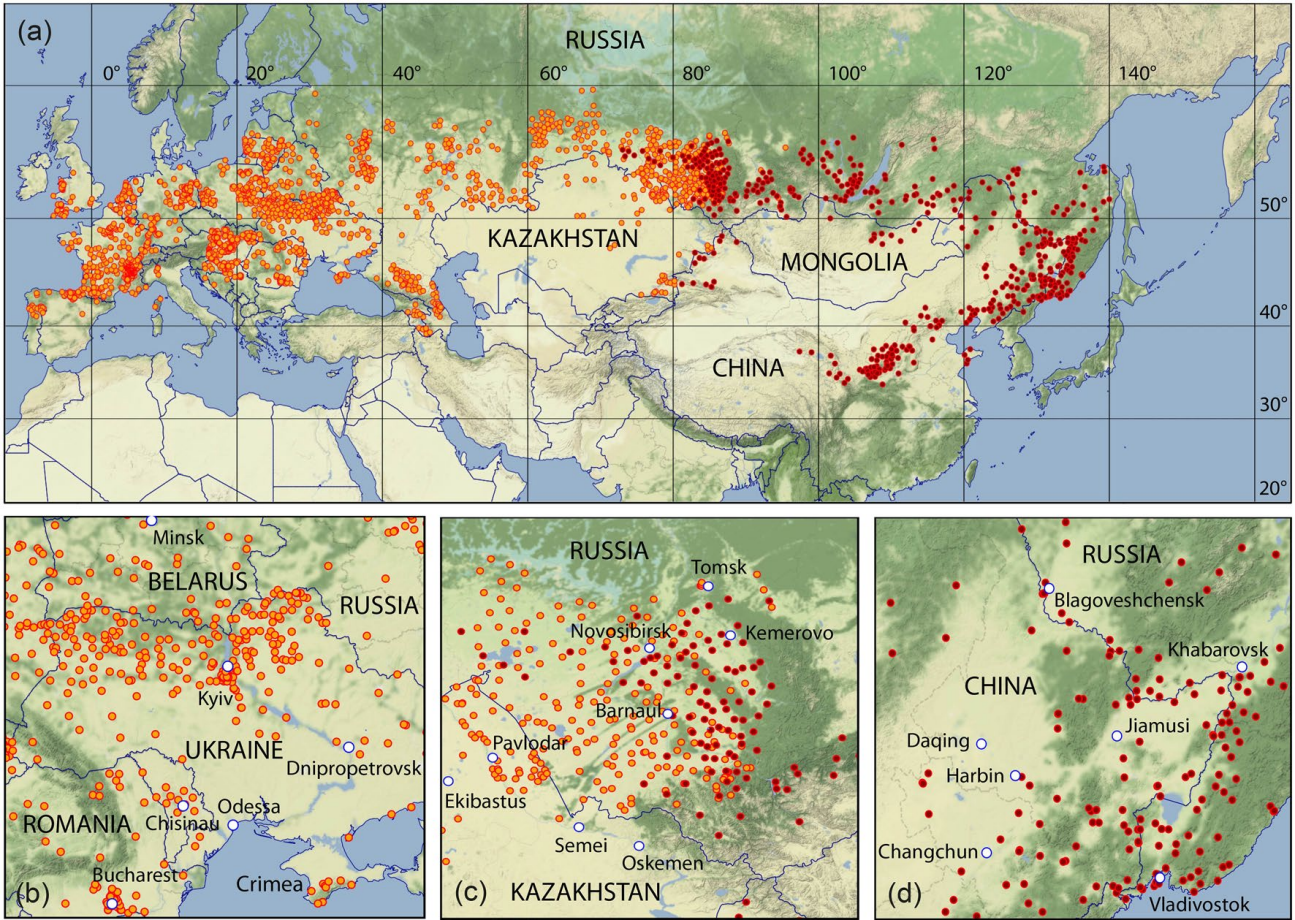
Findings of *Dermacentor reticulatus* (orange) and *Dermacentor silvarum* (red) superimposed on terrain maps. **a** Whole distribution, **b** *D. reticulatus* in Eastern Europe around Kyiv, centred at $30\,^\circ \hbox {E}/49\,^\circ \hbox {N}$, **c** *D. reticulatus* and *D. silvarum* overlapping in the Altai Region around Novosibirsk, centred at $83^\circ \, \hbox {E}/53\,^\circ \hbox {N}$, and **d** *D. silvarum* in the Far East around Harbin, centred at $129^\circ \, \hbox {E}/47\,^\circ \hbox {N}$

Figure 1 depicts a map of the entire distribution areas of the two tick species *D. reticulatus* and *D. silvarum* as well as three high resolution maps of selected regions with findings of these ticks.

The distribution of georeferenced *D. reticulatus* locations within the geographic range of $\hbox {-}9{-}88\,^\circ \hbox {E}/39{-}60\,^\circ \hbox {N}$ is shown in Fig. 1a. Thus, the latitudinal belt, in which *D. reticulatus* occurs in Eurasia, is $5^\circ$ wider than recently described for only Europe (Rubel et al. 2016). The northernmost location of *D. reticulatus* is in Russia at $68.96^\circ \, \hbox {E}/59.73^\circ \, \hbox {N}$, which is confirmed by a second nearby location. The southernmost location might be in Armenia at $46.03^\circ \, \hbox {E}/39.21^\circ \, \hbox {N}$. Both limits were taken from the dataset compiled by Kulik and Vinokurova (1983b). Please note that a total of 5 remote sites from this dataset were removed as part of the reliability check. Two of them were marked by the authors themselves as lying outside the common range of *D. reticulatus*. At 3 sites in Uzbekistan *D. reticulatus* seems to have been confused with the common species *Dermacentor marginatus*. In addition to the reliability check of the tick locations, special attention was paid to closing data gaps in previous distribution maps. These include the first georeferenced locations in Italy, where *D. reticulatus* were collected from vegetation at 8 locations in the Lombardy region of northern Italy (Genchi et al. 2015; Olivieri et al. 2016, 2017). Also in France, new locations expand the documented distribution area of *D. reticulatus*. René-Martellet et al. (2015) published the first findings on the Mediterranean island Corsica and Michelet et al. (2016) on the Atlantic islands Belle-Île-en-Mer, an island off the coast of Western France. In Germany, 30 new locations were documented, including the first findings of *D. reticulatus* at the German Baltic coast in the port of Rostock at $12.14^\circ \, \hbox {E}/54.15^\circ \, \hbox {N}$. This reference confirms the continuous encroachment of *D. reticulatus* to the north during the past 2–3 decades (Paulauskas et al. 2015). It also indicates an almost continuous occurrence of *D. reticulatus* on the Atlantic and Baltic coasts of Europe, reaching from northern Portugal to southern Latvia. An exception is Scandinavia, where no *D. reticulatus* have been found on vegetation (Kjaer et al. 2019), although 21 adult male *D. reticulatus* were found on a migrating Golden jackal (*Canis aureus*) in Denmark (Klitgaard et al. 2017). However, this seems to be a rare case of diversion rather than an indication for a new established occurrence area of *D. reticulatus*. Interesting that only males were found, which might stay on a host for longer than females. Further *D. reticulatus* locations have been documented in the south of England and in Wales (Medlock et al. 2017). The sparse information on the occurrence of *D. reticulatus* in the Western Balkans has been supplemented by 25 locations in Bosnia and Herzegovina, Croatia, and Serbia (Table 1). Many locations are known from Poland, Belarus, and Ukraine (Fig. 1b). Accordingly, *D. reticulatus* is widespread in the entire north of Ukraine, but also in the south of the Crimean peninsula (Hightower 2012). It is also widespread in the European part of Russia from the Caucasus in the south to some 100 km north of Moscow. In the east, the range extends over the north and east of Kazakhstan (Amirova et al. 1989) to Western Siberia, where the Tom river from Tomsk to Kemerovo and further to Novokuznetsk forms the main natural boundary (Fig. 1c). Three remote *D. reticulatus* locations were recorded in the map of Kulik and Vinokurova (1983b), where a location east of Krasnoyarsk at $95.4^\circ \, \hbox {E}/55.6^\circ \, \hbox {N}$ marks the easternmost location. However, these historical locations could not be confirmed in recent studies.

The distribution of georeferenced *D. silvarum* locations in the range of $73{-}140^\circ \, \hbox {E}/34{-}57^\circ \, \hbox {N}$ is also shown in Fig. 1a, where it overlaps with *D. reticulatus* in the region $73{-}88^\circ \, \hbox {E}$, centered around Novosibirsk. The locations digitized from the map of Kulik and Vinokurova (1983a) show that the South Russian distribution area of *D. silvarum* extends to the Pacific coast. More than 20 recent publications confirm the distribution in the Altai region and in the Far East, including the region around Lake Baikal and the north of Mongolia. Among them is the recent study by Obert et al. (2015), which depicts details on the overlapping distribution range of *D. reticulatus* and *D. silvarum* in the Altai region (Fig. 1c). However, most pertinent publications provide *D. silvarum* locations with low accuracy. Therefore, the high accuracy geographical coordinates of the locations provided by Seryodkin et al. (2018) and Kholodilov et al. (2019) must be highlighted. They allow a correct assignment of habitats and climates preferred by *D. silvarum*. In the Far East, the Russian locations seamlessly connect to the Chinese *D. silvarum* locations Jiang et al. (2011). Figure 1d shows that the distribution area of *D. silvarum* extends across the whole of north-eastern China to the capital Beijing and beyond. The eastern and/or southern border forms the Pacific in this region. Chinese *D. silvarum* findings were also reported from the border to the Democratic People’s Republic of Korea. According to the prevailing climate it can be assumed that *D. silvarum* is also endemic in the Democratic People’s Republic of Korea, although there is no evidence for this in the literature. In north-central China, especially in the provinces Heibei, Shanxi, and Shaanxi, *D. silvarum* is also common. Here, the southernmost distribution limit of *D. silvarum* is defined by a location at $108.8^\circ \, \hbox {E}/33.8^\circ \, \hbox {N}$. However, some historical remote locations from the data compilation of Zhang et al. (2019) were not considered in this study as they occur in climates not typical for *D. silvarum* and have not been confirmed, as yet. Locations of *D. silvarum* have also been documented in the north-west of China, in the province of Xinjiang, bordering the distribution area of *D. reticulatus* in Kazakhstan.

Of particular interest is the comparison of the climate adaptations of *D. reticulatus* and *D. silvarum*, which mainly determine the global distribution of each of the two tick species. For this purpose, the tick locations were superimposed on the Köppen–Geiger climate classification map and a frequency distribution of each tick’s occurrence in different climates was compiled.

**Fig. 2:**
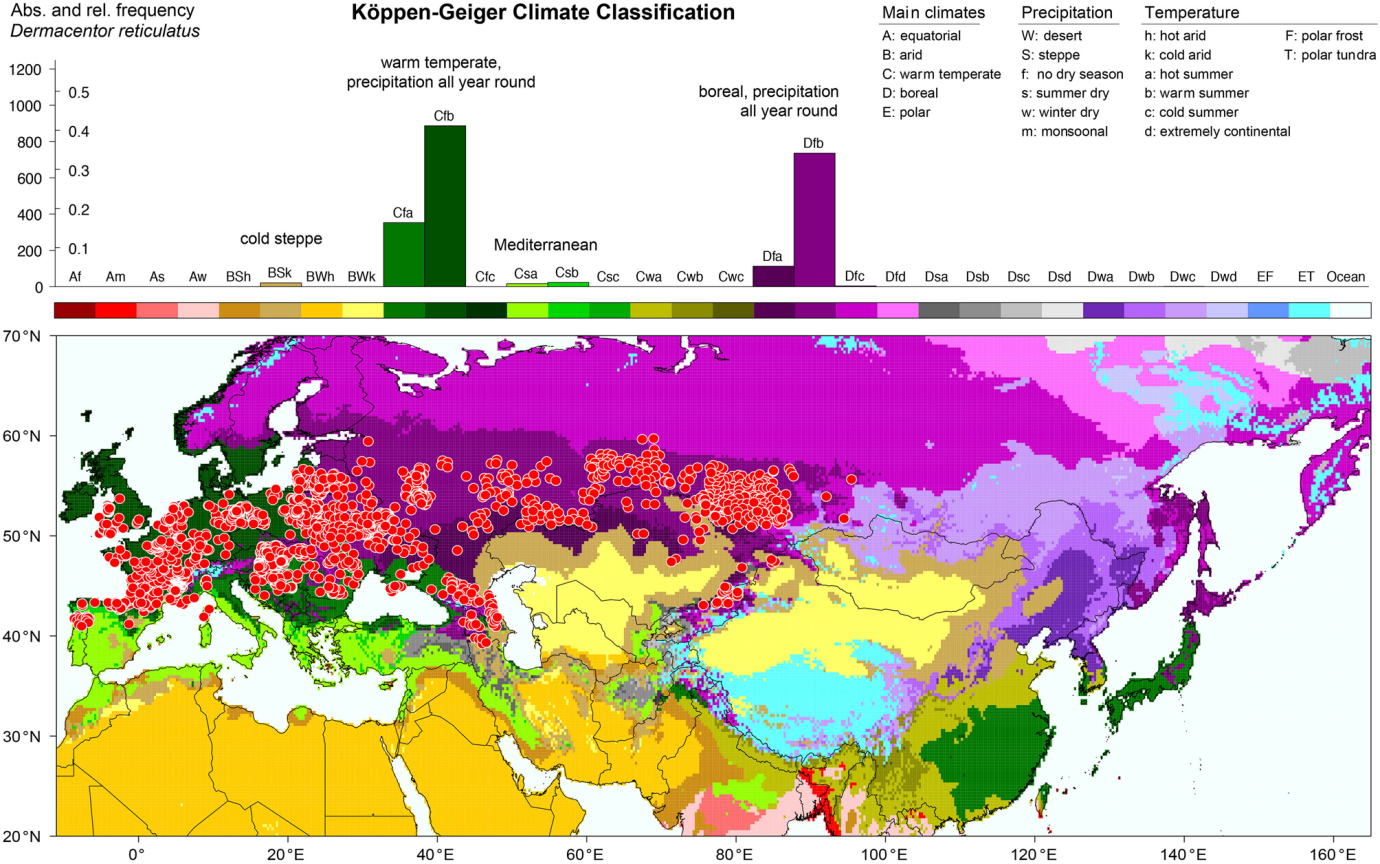
Findings of *Dermacentor reticulatus* superimposed on the map of the Köppen–Geiger climate classification (defined by a three-letter code) and frequency distribution of *D. reticulatus* occurrence. Absolute frequencies depict the number of tick locations, relative frequencies the fraction of tick locations in each climate class. Highest frequencies of *D. reticulatus* occurrence were observed in warm temperate climates with precipitation all year round (Cfa, Cfb) and boreal (continental) climates with precipitation all year round (Dfa, Dfb), both with warm or hot summers

Figure 2 shows the climate classification map together with the Köppen profile for *D. reticulatus*. The latter is a histogram showing the frequency of tick occurrence reported for different climate classes. Two peaks are related to the following climates: warm temperate with precipitation all year round Cf (57 %) and boreal with precipitation all year round Df (40%). Thus, a total of 97% of all *D. reticulatus* locations was reported in these climates, and it is evident that *D. reticulatus* prefers precipitation all year round. In regions with winter temperatures below zero degrees Celsius, *D. reticulatus* thus benefits from a protective snow cover that is hardly available in climates with dry winters. The remaining locations were reported in cold steppes BSk (1%) as well as in Mediterranean climates Csa and Csb (2%). These occurrences of *D. reticulatus* are either due to favourable local climatic conditions or to misidentification. Please note that the Köppen–Geiger climate classification distinguishes between warm temperate (C) and boreal (D) climates only by means of temperature. Warm temperate C climates are defined for a temperature range of $-3\,^{\circ }{\text{C}}< T_{min} < +18\,^{\circ }{\text{C}}$, boreal climates for $T_{min} \le -3\,^{\circ}{\text{C}}$, where $T_{min}$ is the mean temperature of the coldest month of the year (Kottek et al. 2006). A more detailed analysis reveals that warm summers might have a positive influence on the occurrence of *D. reticulatus*. This might also explain the invasion of parts of northern Germany and the Netherlands by this species as well as its encroachment to the north in the Baltic States in the past 2-3 decades. Approximately 75% *D. reticulatus* locations are related to climates with warm summers Cfb and Dfb. Regions with warm summers are defined for a maximal monthly temperature of $T_ {max} < 22\,^{\circ }{\text{C}}$ and at least four months with $T_{mon} \ge 10\,^{\circ }{\text{C}}$ (Kottek et al. 2006). Only 25% of the documented *D. reticulatus* locations are related to climates with hot summers. This makes perfectly sense when taking the necessity for *D. reticulatus* that the development from oviposition to the F1 adult generation has to take place within only one season (Balashov 1972; Kahl and Dautel 2013) and might be one limiting factor for the northernmost distribution of that tick species. This goes also along the fact that *D. reticulatus* is not a forest inhabitant in central Europe, but prefers more open terrains where the soil surface is more sun-exposed (and therefore warmer) in the summer than the forest floor.

**Fig. 3:**
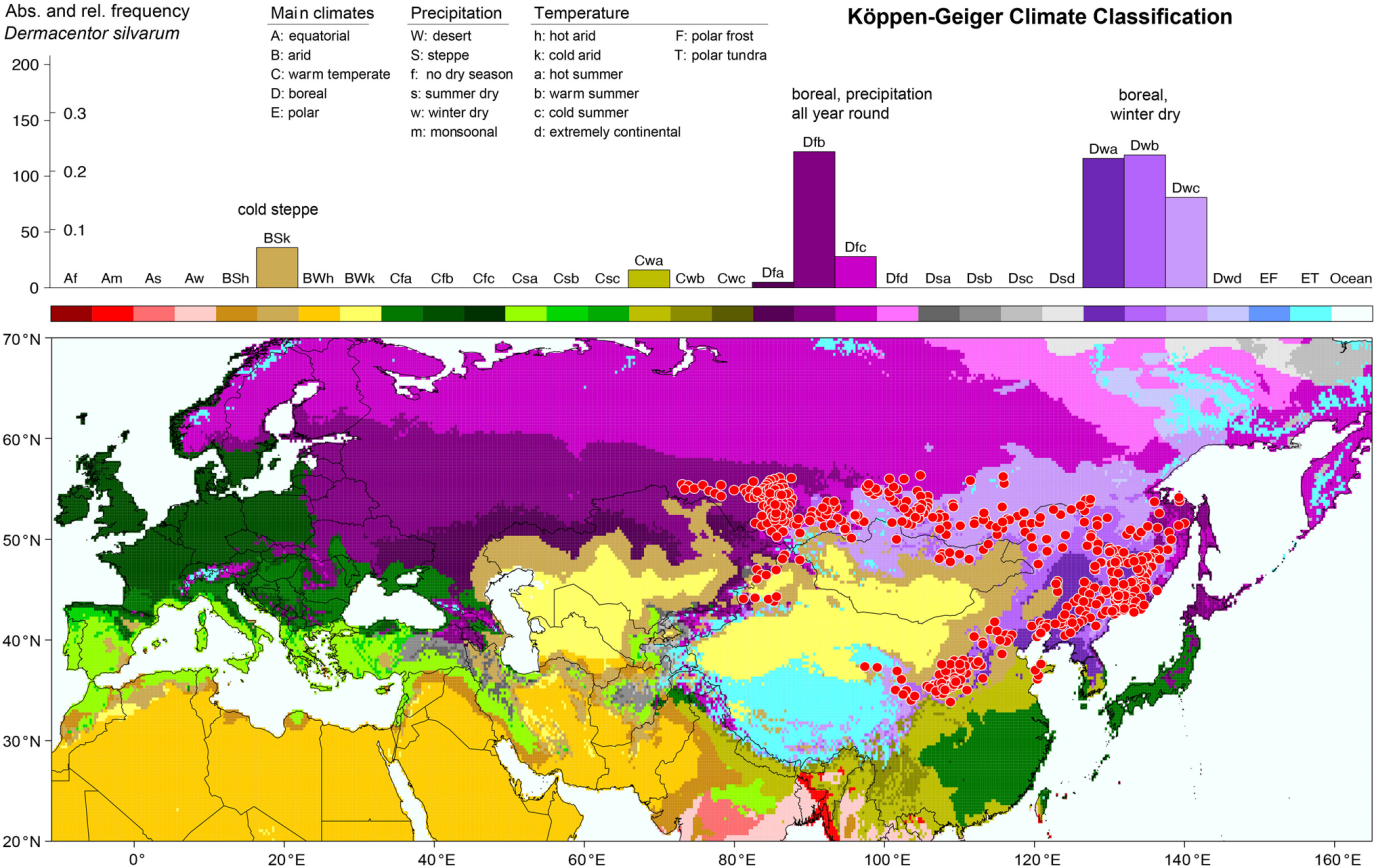
Findings of *Dermacentor silvarum* superimposed on the map of the Köppen–Geiger climate classification and frequency distribution of *D. silvarum* occurrence. Highest frequencies of *D. silvarum* occurrence were observed in boreal (continental) climates with precipitation all year round (Dfb, Dfc) and boreal winter dry climates (Dwa, Dwb, Dwc). Ticks that were found in cold steppe (BSk) and warm temperate winter dry climates with hot summers (Cwa) were mostly collected before 1990, when these locations were still classified as boreal climates (see also Fig. 4)

Figure 3 shows the climate classification map together with the Köppen profile for *D. silvarum* with two major peaks related to the following climates: boreal with precipitation all year round Df (30%) and boreal winter dry (60%). However, *D. silvarum* makes no claims to the summer temperatures, which can range from cool to hot. Climates with hot summers are defined for maximal monthly temperatures of $T_ {max} \ge 22\,^{\circ }{\text{C}}$. Climates with summer temperatures below those defined for hot and warm summers are characterized by cool summers and cold winters if $T_ {min} > -38\,{\circ }{\text{C}}$, otherwise they are called extremely continental (Kottek et al. 2006). Almost 90% of all *D. silvarum* locations are related to boreal Df and Dw climates. The remaining locations were classified as cold steppe BSk (7%) and warm temperate climate with dry winters and hot summers Cwa (3%), exclusively observed in the Chinese distribution area of *D. silvarum*. However, *D. silvarum* does not occur in Cwa climates and also the occurrence in cold steppes is overestimated in the frequency diagram presented in Fig. 3. This can be explained by the rapid climate change in central China. For this purpose, a second map of the Köppen–Geiger climate classification for the period 1956–1980 was calculated and compared with that of 1986–2010.

**Fig. 4:**
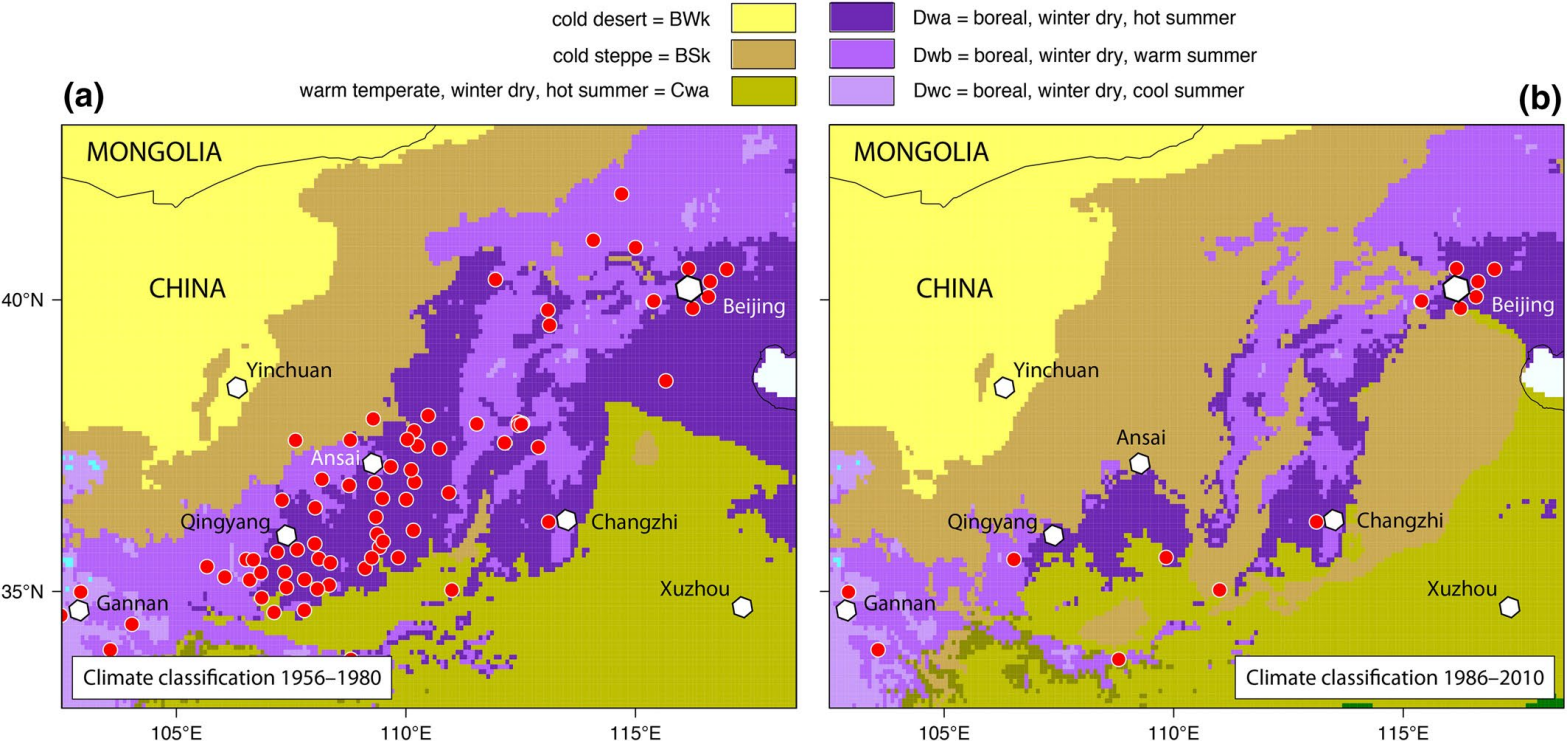
Geographical distribution of *Dermacentor silvarum* (red dots) in north-central and and north-eastern China superimposed on climate maps. **a** Climate classification 1956–1980 with all *D. silvarum* findings and **b** climate classification 1986–2010 with *D. silvarum* findings documented after 1990 (b). Due to climate change, the boreal climates (Dwa, Dwb, Dwc) have been successively replaced by warm temperate climate (Cwa) and arid climates (BWk, BSk), which reduces the habitat suitability for *D. silvarum*

**Fig. 5:**
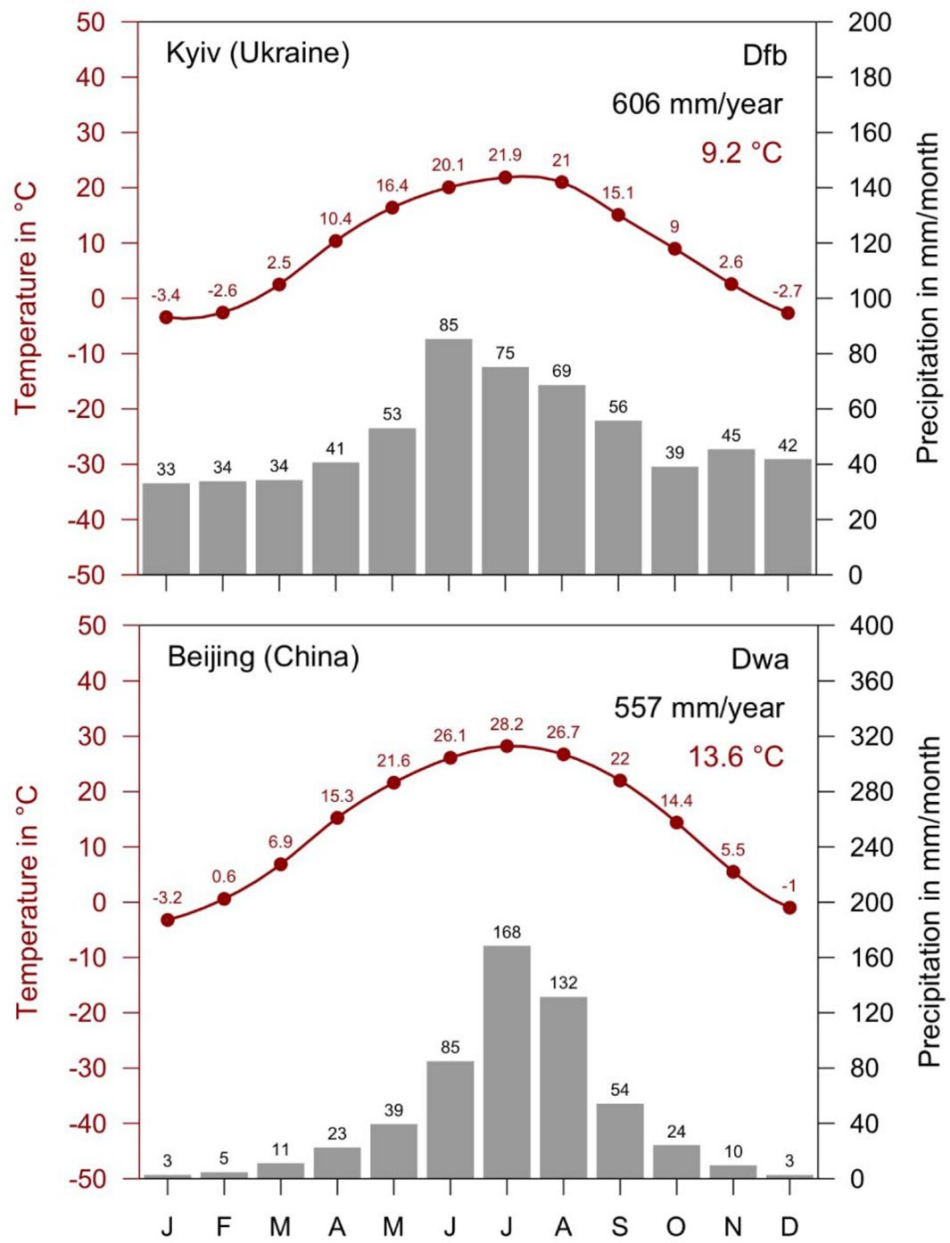
Climate diagrams for the period 1986–2010. Boreal climates with precipitation all year round and warm summer (Dfb) in Kyiv, Ukraine, typical for *Dermacentor reticulatus* locations and winter dry with hot summer (Dwa) in Beijing, China, typical for *D. silvarum* locations

Figure 4 shows a section of these maps centered around the coordinates $110.5^{\circ } \, \hbox {E}/38.0^{\circ }\, \hbox {N}$ southwest of Beijing. The boreal winter dry climates Dwa, Dwb and Dwc, suitable for *D. silvarum*, are surrounded in the north by the cold steppe climate BSk and the desert climate BWk of Inner Mongolia, and in the south by the warm temperate climate Cwa. As climate change progressed, this region became warmer and drier and therefore less suitable for *D. silvarum*. The boreal climates were replaced by cold steppes (BSk) in the north, and by warm temperate climate with hot summers (Cwa) in the south. All those *D. silvarum* found in the Cwa climate of the map representative for the period 1986–2010, were already collected before 1990 in the—at that time still—boreal climates. This is shown by the comparison of the climate map 1956–1980 with all ticks collected from 1954 to present with the climate map 1986–2010, in which only those ticks collected after 1990 are represented. An exception are the 4 *D. silvarum* locations from the Chinese province Shandong ($120^{\circ } \, \hbox {E}/36^{\circ }\, \hbox {N}$, Fig. 3). Here, too, the former Dwa climate was replaced by the Cwa climate, but *D. silvarum* were also found during the period 2015–2017. Regardless, most records in central-northern China are from more than 30 years ago, which is why no exact coordinates are available. In addition, many ticks were collected from animals, which is associated with further uncertainties regarding their exact location. Especially at the borders of climate zones this leads to the fact that *D. silvarum* locations may erroneously be assigned to the arid steppe or warm temperate climates. In this context, the finding of 21 *D. reticulatus* males on a Golden jackal in western Denmark some hundred kilometres away from the nearest known place of occurrence of that species (Klitgaard et al. 2017) is very interesting. This example shows that such cases may happen but that it is necessary to carefully differentiate between the core distribution of a species where it goes through the whole life cycle and other areas—possibly with unsuitable climate—where that species may occur sporadically in limited numbers for a short period of time. In any case, however, it can be stated that *D. silvarum* is better adapted to arid regions as well as to hot summers than *D. reticulatus*. This can also be seen when comparing climate diagrams typical of *D. reticulatus* locations in Kyiv and *D. silvarum* locations in Beijing (Fig. 5).

**Table 3:** Detection of tick-borne pathogens or their DNA/RNA in adult questing *Dermacentor silvarum*

Pathogen	Disease	Method	Country	References
*Viruses*				
Blacklegged tick phlebovirus		D	China	Meng et al. (2019)
Deer tick *Mononegavirales*-like virus		D	China	Meng et al. (2019)
Lymphocytic choriomeningitis virus	Lymphocytic choriomeningitis	D	China	Zhang et al. (2018)
Powassan virus	Encephalitis	D	Russia	Leonova et al. (2009)
Tick-borne encephalitis virus	Tick-borne encephalitis	P	Russia	Zhmaeva and Pchelkina (1967b)
		D	Russia	Shchuchinova et al. (2015)
		D	Russia	Verkhozina et al. (2017)
		D	Russia	Pukhovskaya et al. (2018)
		D	Russia	Kholodilov et al. (2019)
		D	China	He et al. (2018)
*Bacteria*				
*Anaplasma phagocytophilum*	Human granulocytic anaplasmosis	D	Russia	Doroshchenko et al. (2019)
		D	China	Jiang et al. (2011)
		D	China	Wei et al. (2016)
		D	China	Cao et al. (2006)
*Borrelia burgdorferi* s.l.	Lyme borreliosis	D	Russia	Leonova et al. (2015)
		D	Russia	Dragomeretskaya et al. (2018)
		D	Russia	Pukhovskaya et al. (2019)
*B. miyamotoi*		D	Russia	Dragomeretskaya et al. (2018)
*Coxiella burnetii*	Q fever	P	Russia	Zhmaeva and Pchelkina (1967a)
*Ehrlichia chaffeensis/E. muris-FL*	Human monocytotropic ehrlichiosis	D	Russia	Leonova et al. (2015)
*Francisella tularensis*	Tularaemia		Russia	Olsufiev and Petrov (1967)
			Russia	Balashov (1972)
			Russia	Filippova (1997)
			Russia	Kudryavtseva et al. (2018)
		D	China	Zhang et al. (2008)
*Rickettsia raoultii*	TIBOLA/DEBONEL^a^	D	China	Tian et al. (2012)
		D	China	Wen et al. (2014)
		D	China	Liu et al. (2016)
		D	China	Han et al. (2018)
		D	Russia	Mediannikov et al. (2008)
		D	Russia	Igolkina et al. (2018)
*R. heilongjiangensis*	Far Eastern spotted fever	P	China	Sun et al. (2015) (review)
*R. sibirica*	North Asian/Siberian tick typhus	P	Russia	Korshunova (1967)
		D	Russia	Shulunov et al. (2007)
*R. slovaca*		D	China	Tian et al. (2012)
*Rickettsia* sp. DnS14	TIBOLA/DEBONEL^a^	D	Russia	Shulunov et al. (2007)
*Rickettsia* sp. DnS28	TIBOLA/DEBONEL^a^	D	Russia	Shulunov et al. (2007)
*Candidatus* R. tarasevichiae	Unnamed rickettsiosis	D	Russia	Igolkina et al. (2018)
*Candidatus* R. gannanii Y27		D	China	Han et al. (2018)
*Candidatus* R. tibetani		D	China	Han et al. (2018)
*Piroplasmorida (Protozoa)*				
*Babesia venatorum*	Babesiosis	D	China	Jia et al. (2018)
*B. motasi*-like	Babesiosis	D	China	Niu et al. (2016)

Method indicates isolation of the tick-borne pathogen (P) or detection of its DNA/RNA (D)

^a^Tick-borne lymphadenopathy/*Dermacentor*-borne necrosis erythema and lymphadenopathy

In summary, it was shown that *D. reticulatus* occurs in warm temperate or boreal climates with precipitation all year round and warm summers. In contrast, *D. silvarum* occurs preferentially in boreal (continental) climates, whereby there must only be sufficient precipitation in the summer half-year. The fact that *D. silvarum* is particularly well adapted to cold winter temperatures is shown by the lower lethal temperatures (50% survival) for larvae and nymphs of approximately $-16^{\circ }\,\hbox{C}$ and for adults of $-20^{\circ }\, \hbox {C}$. The temperatures, at which *D. silvarum* body fluids spontaneously freeze, was determined to be $-20^{\circ }\, \hbox {C}$ and $-24^{\circ }\, \hbox {C}$, respectively (Wang et al. 2017). The occurrence of *D. silvarum* in dry steppes or in warm temperate climates can be frequently attributed to the fact that these climates were boreal at the time the historical tick locations were reported. Related frequencies of *D. silvarum* occurrence (BSk and Cwa climates) in the histogram in Fig. 3 are therefore mainly statistical artefacts. In the interpretation of the absolute and relative abundances of the climates suitable for ticks, it must also be considered that these could be biased due to the presence or absence of local tick research groups.

Because the geographical distribution of vector tick species is particularly important in connection with tick-borne diseases, an overview of pathogens detected in adult questing *D. reticulatus* ticks was compiled by Rubel et al. (2016). That table should be extended by TBE virus repeatedly isolated from adult *D. reticulatus* ticks in an endemic area in Germany (Chitimia-Dobler et al. 2019). In this context, also the study on acute canine babesiosis in Belgrade (Serbia) is of interest (Janjić et al. 2019). Table 3 provides a summary of the pathogens found in questing *D. silvarum*. Also in *D. silvarum*, TBE virus or its RNA was detected in both China (He et al. 2018) and Russia (Kholodilov et al. 2019). In addition, 4 other viruses and numerous pathogenic bacteria and protozoa have been found in *D. silvarum*. However, the finding of any tick-borne pathogens in questing ticks is no proof of vector competence but at least confirms the state of a carrier. Without proven capability of transmission the vector function of a given tick species for a given pathogen is not substantiated (Kahl et al. 2002). Mere determination of the carrier status of field-collected questing ticks is only a very first step to indicate vector competence.

Table 4 summarizes the results of studies which demonstrated vector competence of *D. reticulatus* for various pathogens, i.e. successful transmission experiments with hosts. For example, the studies by Koz̆uch and Nosek (1971), Alekseev and Chunikhin (1991) and Belova et al. (2013) confirmed the role of *D. reticulatus* as a vector for the TBE virus. The same applies to *R. sibirica*, the causative agent of North Asian/Siberian tick typhus (Korshunova 1967). Further successful transmission experiments of pathogens from infected ticks to exposed hosts were carried out for the Palma virus (Labuda et al. 1997), *Coxiella burnetii* (Zhmaeva and Pchelkina 1967b), *Anaplasma marginale* (Zivkovic et al. 2007), and *B. canis* (Varloud et al. 2018). According to Balashov (1972) and the European Centre for Disease Prevention and Control (www.ecdc.europa.eu/en/tularaemia/facts), *D. reticulatus* is also a proven vector of *Francisella tularensis*, the causative agent of tularaemia. Both transstadial survival and transovarial transmission was demonstrated for *Borrelia afzelii* (Rudakova et al. 2005), for *Anaplasma* sp. Omsk (Krasikov et al. 2007), and for *Brucella* sp. (no mention of the species!) (Rementsova and Khrushcheva 1967) in *D. reticulatus*, but this alone does not mean that this tick species can transmit these agents to hosts.

**Table 4:** Vector competence of *Dermacentor reticulatus* based on the results of transmission studies (tick to host transmission TH, transovarial transmission TO, transstadial survival TS, and tick stages larva L, nymph N, adult A)

Pathogen	Disease	Transmission	Tick stage	References
Palma virus		TH (white mice, co-feeding)		Labuda et al. (1997)
Omsk haem. fever virus		TH (human), TO, TS	N, A	Růžek et al. (2010) (review)
TBE virus	Tick-borne encephalitis	TH (white mice)	N	Koz̆uch and Nosek (1971)
		TO, TS	L, N, A	Zhmaeva and Pchelkina (1967b)
		TO, TS	L, N, A	Naumov et al. (1980)
		TO, TS	L, N, A	Belova et al. (2013)
		TS	N, A	Karbowiak et al. (2016)
		TH (human)	A	Valitskaya et al. (2016)
		TH (white mice, co-feeding)	A	Ličková et al. (2020)
*Anaplasma marginale*	Bovine anaplasmosis	TH (calfs)	A (male)	Zivkovic et al. (2007)
*Coxiella burnetii*	Q fever	TH (guinea pigs), TO, TS	L, N, A	Zhmaeva and Pchelkina (1967a)
*Rickettsia raoultii*	TIBOLA/DEBONEL^a^	TO, TS		Samoylenko et al. (2013)
		TH (human)	A (male)	Földvári et al. (2013)
*R. slovaca*	TIBOLA/DEBONEL^a^	TH (human)	A	Raoult et al. (2002)
		TH (human)	A (male)	Földvári et al. (2013)
*R. sibirica*	N. Asian/Siberian tick typhus	TH (white mice), TO, TS	A	Korshunova (1967)
*Babesia caballi*	Equine piroplasmosis	TH, TO	N, A	Friedhoff (1988) (review)
*B. canis*	Canine babesiosis	TH, TO	A	Friedhoff (1988) (review)
		TH (dog)	A	Varloud et al. (2018)

Review papers are given when historical papers were not available

^a^Tick-borne lymphadenopathy/*Dermacentor*-borne necrosis erythema and lymphadenopathy

Table 5 summarizes successful pathogen transmission studies with *D. silvarum*. The number of studies is much smaller than that for *D. reticulatus*. TBE virus (Zhmaeva and Pchelkina 1967b), *Rickettsia sibirica* (Korshunova 1967), and *Babesia caballi* have been shown to be transmitted by *D. silvarum*. Transstadial survival in *D. silvarum* was found for Powassan virus (Kruglyak and Leonova 1989), for *Rickettsia raoultii* (Samoylenko et al. 2013) and for *Anaplasma* sp. Omsk (Krasikov et al. 2007). Transovarial transmission by *D. silvarum* was demonstrated for *R. raoultii* (Samoylenko et al. 2013) and for *Anaplasma* sp. Omsk (Krasikov et al. 2007). Although these studies indicate some susceptibility of *D. silvarum* for each of those three agents, the proof of its vector competence for them is still missing. No vector competence of *D. silvarum* could be demonstrated for *B. garinii* (Sun and Xu 2003). Larval and nymphal *D. silvarum* efficiently acquired spirochaetes from infected hosts, but spirochaetes could no longer be detected from engorged larval and nymphal ticks 1–2 weeks after repletion, and there was no evidence of transstadial passage to the resulting nymphs or adults.

**Table 5:** Vector competence of *Dermacentor silvarum* based on the results of transmission studies (tick to host transmission TH, transovarial transmission TO, transstadial survival TS, and tick stages larva L, nymph N, adult A)

Pathogen	Disease	Transmission	Tick stage	References
TBE virus	Tick-borne encephalitis	TH (guinea pigs), TO, TS	L, N, A	Zhmaeva and Pchelkina (1967b)
*R. sibirica*	N. Asian/Siberian tick typhus	TH (white mice)	A	Korshunova (1967)
*Babesia caballi*	Equine piroplasmosis	TH, TO	N, A	Friedhoff (1988) (review)

Review papers are given when historical papers were not available

## Conclusions and outlook

*Dermacentor reticulatus* has been in the focus of research for decades, because it is a long-known proven vector of TBE virus and *B. canis* (Table 4). *Dermacentor silvarum* is a proven vector of TBE virus, *R. sibirica*, and *B. caballi* (Table 5). Both *Dermacentor* species can often be found in considerable numbers in green spaces and parks of large cities. To summarize the current knowledge on the distribution of *D. reticulatus* and *D. silvarum*, comprehensive datasets of georeferenced tick locations were compiled to draw the first geographical maps covering their entire distribution ranges. Although these data collections are one of the most extensive, they have some weak points. For example, there are no data for the Korean Peninsula and for Sakhalin (Kiefer et al. 2010), although *D. silvarum* should occur in both regions. Furthermore, many geographical descriptions do not include coordinates, which is why a high number of locations is known only with low accuracy (Tables 1, 2), especially in Asia reported in the Chinese and Russian literature. Only in recent scientific papers (Jiang et al. 2011; Kholodilov et al. 2019) exact geographic coordinates were given. This should be standard in future field studies.

Another problem that should be solved in future studies are reported locations of tick species, which might be unreliable. Especially, determination of *Dermacentor* species in the Far East before 1980 are questionable. Concerning historical data, Yamaguti et al. (1971) stated in their book *Ticks of Japan, Korea, and the Ryukyu Islands* that they have not attempted to determine the species of the genus *Dermacentor*, since there is very little information. The data collection of Zhang et al. (2019) going back to 1954 lists 29 *D. reticulatus* locations in the Chinese province Shaanxi, which appear questionable. The region southwest of Beijing, shown in Fig. 4, is much too dry for *D. reticulatus* in the winter half-year. Since these sites are more than 2000 km away from the nearest endemic areas of *D. reticulatus* in Kazakhstan and Russia and were not confirmed by independent working groups, they were not used here. However, they have already been adopted in several literature reviews, which gives the impression of confirmed *D. reticulatus* occurrences in China. This problem can only be finally clarified by a verification of *D. reticulatus* samples from Shaanxi.

Future studies will also have to pay more attention to the assignment of the correct climate to the tick locations. It should be noted that climate data do not have the same high accuracy in large areas of Central Asia, especially in arid and mountainous regions, as in Europe. The reason for this is the climate measurement network, which is not very dense in sparsely populated areas. Most of the *Dermacentor* locations, which were identified in the climate statistics as arid, could therefore be misclassified due to uncertainties of geographic allocation as well as climate classification. In addition, there is climate change, which also contributes to an uncertainty in determining the suitable climates for the 2 tick species *D. reticulatus* and *D. silvarum* (Fig. 4). In subsequent studies, the tick locations must therefore be selected according to their date in order to take the exact climate requirements into account. Only then can the georeferenced tick locations be used by species distribution models (SDMs) to determine the impact of climate change on the future distribution of tick species. Previous predictions with SDMs have neglected climate change in the past, which is why they often provide unrealistic future tick distributions.

Although tick-borne agents have often been found in questing adult *D. reticulatus* and *D. silvarum* (Table 3), experimental transmission studies are still largely lacking. Linking proved vector competence for certain pathogens and large-scale tick occurrence with tick-borne diseases would strongly support the work of public health authorities. To the authors’ knowledge, there is only one paper on this topic. For China, Sun et al. (2017) compiled a national TBE map based on an SDM that uses the distribution of the TBE vectors (*I. persulcatus*, *D. silvarum*, *H. concinna*, and other tick species) as additional predictors. In order to be able to create a similar map for the entire TBE endemic area in Eurasia, the data sets presented here must be expanded at least by the main TBE vectors *I. ricinus* and *I. persulcatus*. The Central European TBE map, which is based on TBE positive ticks and endothermic mammals (Walter et al. 2020), could also be extended to the entire TBE distribution area with such tick data.
